# PD133 Exploring Real-World Data Based Financing Models For Lung Cancer Immunotherapy In The Czech Republic

**DOI:** 10.1017/S0266462324003702

**Published:** 2025-01-07

**Authors:** Adéla Bártová, Barbora Říhová

## Abstract

**Introduction:**

In alignment with recommendations from the Czech Society for Oncology, immunotherapy is gaining prominence in managing metastatic lung cancer. Public health insurance in the Czech Republic covers immunotherapy for defined categories of these malignancies. Our study aimed to evaluate the impact of introducing performance-based risk-sharing agreements (PBRSA) on budgetary considerations for immunotherapy treatment.

**Methods:**

In collaboration with the Masaryk Memorial Cancer Institute in Brno, we conducted a retrospective analysis of 127 patients with lung cancer who were treated with immunotherapy (42 received nivolumab) between 2018 and 2022. We explored the reimbursed indications for pembrolizumab, nivolumab, atezolizumab, and durvalumab. Real-world progression-free survival (PFS) data from the medical records were compared with PFS data from randomized controlled trials. Patients were classified as either successfully or unsuccessfully treated according to the PFS threshold established in the comparator arm of the respective trials. Additionally, we explored a hypothetical scenario involving the potential implementation of PBRSA depending on the level of outcome achieved.

**Results:**

In patients with advanced lung cancer who had received prior chemotherapy, nivolumab succeeded in 29 patients but failed to meet the defined success threshold in 13 patients. Unsuccessful cases incurred an average cost of EUR9,300 per patient over a median treatment period of 1.7 months. In contrast, the cost of successful treatment exceeded EUR29,700 per patient, which was sustained for a median treatment duration of 5.5 months. Manufacturers could cover up to 66 percent of the cost associated with unsuccessful treatment in the 13 patients, which would exceed EUR41,000. This approach might cover the expenses for one additional patient. The same calculation was performed for all other checkpoint inhibitors.

**Conclusions:**

The analysis emphasizes the vital role of risk-sharing agreements in enhancing the affordability and sustainability of high-cost advanced therapies. Discrepancies between real-world clinical data and registration studies challenge full reimbursement sustainability. By redistributing financial responsibility, PBRSA alleviate costs for insurers and simplify market entry for manufacturers, contributing to a dynamic and inclusive healthcare landscape.

